# Using the Diamagnetic Coefficients to Estimate the Reduced Effective Mass in 2D Layered Perovskites: New Insight from High Magnetic Field Spectroscopy

**DOI:** 10.3390/ijms232012531

**Published:** 2022-10-19

**Authors:** Mateusz Dyksik

**Affiliations:** Department of Experimental Physics, Faculty of Fundamental Problems of Technology, Wroclaw University of Science and Technology, 50-370 Wroclaw, Poland; mateusz.dyksik@pwr.edu.pl

**Keywords:** effective mass, magnetic field, diamagnetic coefficient, 2D perovskites, Landau levels

## Abstract

In this work, the current state of research concerning the determination of the effective mass in 2D layered perovskites is presented. The available experimental reports in which the reduced effective mass μ has been directly measured using magneto-absorption spectroscopy of interband Landau levels are reviewed. By comparing these results with DFT computational studies and various other methods, it is concluded that depending on the approach used, the μ found spans a broad range of values from as low as 0.05 up to 0.3 m${}_{e}$. To facilitate quick and reliable estimation of μ, a model is proposed based solely on the available experimental data that bypass the complexity of interband Landau level spectroscopy. The model takes advantage of the μ value measured for (PEA)${}_{2}$PbI${}_{4}$ and approximates the reduced effective mass of the given 2D layered perovskites based on only two experimental parameters—the diamagnetic coefficient and the effective dielectric constant. The proposed model is tested on a broad range of 2D layered perovskites and captures well the main experimental and theoretical trends.

## 1. Introduction

The effective mass of the charge carriers (m${}^{*}$) is a fundamental parameter derived from the band theory of solids, which describes how interaction with the lattice changes the mass of the charge carriers, with respect to the free electron mass. The effective mass influences other important physical parameters, such as the charge carrier mobility and diffusion length. It plays a crucial role in semiconductor physics—in the simplest Drude model of electronic transport, it determines the maximum obtainable charge carrier velocity $v∼\frac{1}{m^{*}}$, thereby determining the ultimate speed of integrated circuits [1]. Furthermore, it is linked directly to the exciton binding energy, thus determining the practical application of semiconducting materials in either light absorbers (solar cells) or emitters. Since it reflects the dispersion of bands and can be calculated from the known band structure, experimental determination of the effective mass is a crucial test of the theoretical electronic structure.

Experimentally, the effective mass is traditionally determined from cyclotron resonance, which probes the electronic transitions between intraband Landau levels [2]. In an external magnetic field, the electrons (holes) move on closed-loop trajectories within the plane normal toward the magnetic field vector **B**. When the incident electromagnetic radiation of frequency ω equals the electron (hole) angular frequency $\omega_{c}$, the corresponding effective mass can be determined via $\omega_{c}=\frac{eB}{m_{e,h}}$, without any additional input parameters (*e*-elementary charge). This approach was successfully employed to determine the effective masses of silicon, germanium [3], and many other semiconducting materials [4,5]. The cyclotron resonance technique can be extended to probe the transitions between interband Landau levels in the magneto-absorption experiment. In this case, the optical transition is detected between the valence and conduction band states; thus, the reduced effective mass μ is determined. The magneto-absorption experiment was successfully used to determine the mass of charge carriers in traditional fully inorganic bulk semiconductors [6,7,8] and lower dimensional variants [6,9]. This method was also successfully used to determine the reduced effective mass in the family of 3D organic–inorganic halide perovskites [10,11,12,13] and, more recently, for its derivatives, namely 2D layered perovskites [14,15].

Two-dimensional layered perovskites are organic–inorganic hybrid structures consisting of metal–halide octahedral units arranged in a planar form in which each plane is structurally separated by large (usually hydrophobic) organic cations. Such a configuration results in a material that is much more resistant to ambient conditions than its 3D counterparts [16,17,18], making it ideal for applications in future opto-electronic devices [19,20,21,22]. The large organic molecules not only enhance the stability of the resulting 2D material but also provide control over the dielectric confinement and crystal structure [23,24,25,26,27,28], making them an intriguing material for fundamental investigation and band structure engineering.

Starting from the very early reports concerning the excitonic and electronic properties of 2D perovskites, magneto-spectroscopy [29] has been the experimental tool of choice, revealing many intriguing aspects of exciton physics in these materials. An analysis of the main optical feature’s behavior with an increasing magnetic field demonstrated that the optical response is dominated by strongly bound two-dimensional excitons with transitions that shift diamagnetically (energy shift $∼B^{2}$) up to B=40 T [29,30]. In addition, one of the first high magnetic field measurements suggested that the multitude of high-energy features observed in the absorption spectrum is related to phonon-replicas [30,31], which was confirmed by the very similar diamagnetic coefficients and validated by later experiments [32]. Most importantly, a model for the exciton manifold in 2D layered perovskites was proposed based on high magnetic field spectroscopy [30] and followed by subsequent studies [33,34]. At the band edge, four excitonic states were suggested: a triple-degenerate bright state and an exchange-interaction split-off dark state. This model was used to understand the emission spectrum of many 2D perovskites [33,34,35], suggesting that the bright–dark splitting is of the order of tens of meV. A very recent report completes the exciton’s fine structure picture, showing that indeed the lowest-lying excitation in this material system is a dark exciton [36].

Although the early high magnetic field studies made it possible to shape the current state of knowledge concerning excitons in 2D layered perovskites, some parameters, such as the effective mass, remained inaccessible; the effective mass can be unequivocally determined only from Landau level spectroscopy. This is surprising considering that the magneto-spectroscopy of 2D layered perovskites preceded similar investigations of 3D perovskites [37], for which direct measurement of the reduced effective mass has been very successful [10,11,12,13].

The overall complexity involved in observing the optical transitions between Landau levels in the magneto-absorption experiment on 2D layered perovskites results from both the structural quality of the polycrystalline films (low carrier mobility) and the spectral region of the interband Landau level transitions. Most of the Pb-based 2D perovskites are characterized by a large exciton binding energies of hundreds of meV and bandgaps around 3 eV [24,38]. Thus, in order to observe the free carrier transition between Landau levels, a high magnetic field is required to overcome both the Coulomb attraction and the low mobility of the charge carriers. The optical measurements in the high magnetic field facilities (B≥60 T) are performed using optical fiber-based insets with poor spectral sensitivity beyond 3 eV. As a result, direct determination of the reduced effective mass from the magneto-absorption experiment is limited to only a few reports [14,15], demonstrating the need for an alternative method to determine the effective mass.

As a remedy, different approaches were pursued based on the more accessible diamagnetic coefficient. For bulk and 2D systems, the diamagnetic coefficient is a measure of the root-mean-square extension of the wave function (in the plane normal to the magnetic field vector **B**), which, in turn, is proportional to the exciton Bohr radius and binding energy [2]. Thus, the diamagnetic coefficient can be used to determine the effective mass if the form of the potential is known. Early investigations assumed a 2D exciton model with a Coulomb potential [31]. Subsequently, the image charge method was employed to take into account the dielectric contrast between the organic and inorganic materials [33]. In addition, more advanced models, including complex DFT computing of the dielectric profiles, were employed to determine the reduced effective mass based on the measured diamagnetic coefficient [38]. Unfortunately, the value of the effective mass determined from the various methods spans a broad range.

In this work, the current state of knowledge regarding the effective mass in 2D layered perovskites is reviewed. The available experimental reports in which the reduced effective mass μ was directly measured by means of magneto-absorption spectroscopy are discussed. Comparing these values with those of the masses computed from the band dispersion reveals a large disparity in the reported effective mass in the literature. In the latter section, an alternative approach is developed to facilitate a reliable approximation of μ. A model is formulated to approximate the reduced effective mass based on its dependence on the diamagnetic coefficient $c_{0}$ and the effective dielectric constant $\epsilon_{\infty }$. Since all three parameters (μ, $c_{0}$, $\epsilon_{\infty }$) for the model sample (PEA)${}_{2}$PbI${}_{4}$ are experimentally well known, the proportionality factor between them is determined, which can then be used to determine the reduced effective mass in various 2D layered perovskites, including (PEA)${}_{2}$PbBr${}_{4}$.

**Figure 1:**
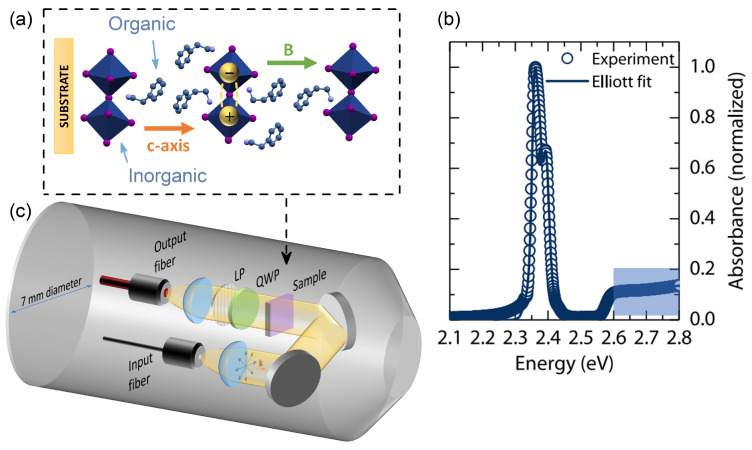
(**a**) Structure of 2D (PEA)${}_{2}$PbI${}_{4}$ layered perovskite. Arrows indicate the direction of the magnetic field vector **B** and **c**-axis vector with respect to the substrate and inorganic planes (Faraday geometry). (**b**) Low-temperature absorption spectrum of (PEA)${}_{2}$PbI${}_{4}$. Reprinted with permission from Neutzner et al. [43]. Copyright 2022 by the American Physical Society. (**c**) Schematic drawing of the high magnetic field inset tip. QWP—quarter-wave plate, LP—linear polarizer, **k**—wave vector of the probing light.

Two-dimensional layered perovskites consist of thin inorganic slabs of lead (tin)–halide octahedra, separated by large organic molecules (spacers) [39,40,41], as schematically illustrated in Figure 1a. They are often described as ideal quantum wells, in which the excitons are confined in the central metal–halide slab, while the organic spacers act as barriers [38,42] (Figure 1a). A typical absorption spectrum of the prototypical 2D layered perovskite (PEA)${}_{2}$PbI${}_{4}$ ([C${}_{6}$H${}_{5}$(CH${}_{2}$)${}_{2}$NH${}_{3}$]${}_{2}$PbI${}_{4}$, PEA—phenylethylammonium) is presented in Figure 1b. The spectrum is dominated by absorption of the 1s exciton at ∼2.35 eV. An increase in absorption is also observable at a higher energy of ∼2.6 eV, which is attributed to the band gap [14,43].

For (PEA)${}_{2}$PbI${}_{4}$, it was possible to directly measure the reduced effective mass μ via observation of the interband optical transitions between Landau levels in the magneto-absorption experiment [14,15]. To do so, a pulsed magnetic field with a maximum strength of B=65 T was used. In short, a semi-transparent thin film was placed on the field center of the high magnetic field transmission inset, which is schematically presented in Figure 1c. The broadband white light was provided by a Xe lamp and sent to the sample using a 100 μm core input fiber (see Figure 1c). The transmitted signal was collected by a lens, coupled to the 400 μm core of the output fiber, and analyzed with a spectrometer.

**Figure 2:**
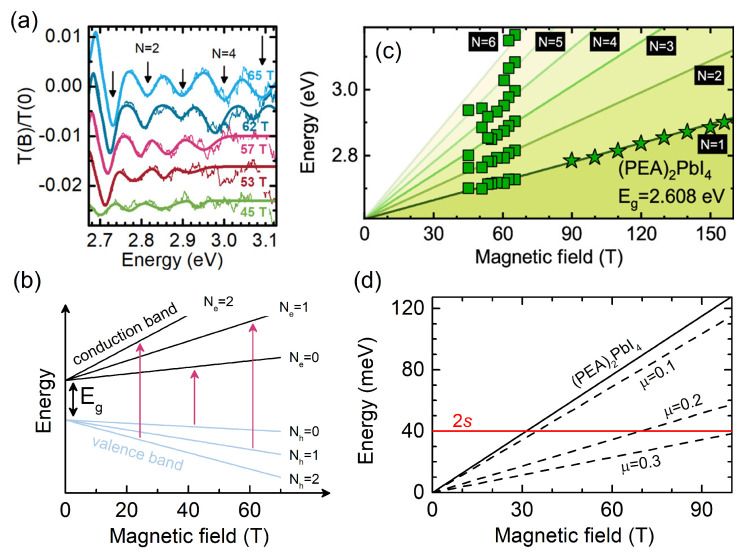
(**a**) Transmission ratio spectra T(B)/T(0) of (PEA)${}_{2}$PbI${}_{4}$ at selected field strengths. (**b**) Schematic showing the evolution of Landau levels with increasing magnetic field. Arrows indicate the allowed interband optical transitions between Landau levels in the valence and conduction band, i.e., $N_{h}=N_{e}$. (**c**) Energy of the interband landau level transitions as a function of the magnetic field for (PEA)${}_{2}$PbI${}_{4}$. Solid lines represent fits with Equation (1). (**d**) Cyclotron energy dependence on the magnetic field strength for several reduced effective mass values and (PEA)${}_{2}$PbI${}_{4}$ (solid black). The 2*s* state binding energy of (PEA)${}_{2}$PbI${}_{4}$ is indicated [44]. Panels (**a**,**c**) are reprinted (adapted) with permission from Dyksik et al. [14]. Copyright 2022 American Chemical Society.

Figure 2a shows a typical transmission ratio spectrum i.e., T(B)/T(0) for (PEA)${}_{2}$PbI${}_{4}$ in the above bandgap spectral range (indicated in Figure 1b with a blue rectangle). A number of new absorption features are visible, as indicated by the arrows. At a given magnetic field, these new absorption lines are equally spaced in energy—a fingerprint of the interband optical transitions between Landau levels.

In a two-dimensional system, as in quantum wells, the optical transitions are allowed only between the valence and conduction band Landau levels of the same orbital quantum number $N=N_{e}=N_{h}$, as schematically depicted by the arrows in Figure 2b. The energy $E_{N}$ of the given transition is defined as:

(1) $$E_{N}=E_{g}+\left(N+\frac{1}{2}\right)\frac{\hbar eB}{\mu }$$

where $E_{g}$ is the bandgap energy, *N* is the Landau quantum number, and μ is the reduced effective mass. Since the magnetic field restricts (quantizes) the in-plane motion to closed-loop trajectories, and there is no degree of freedom in the motion along the magnetic field vector due to the quantum confinement (2D nature), the density of the state of each Landau level is a delta function. In the ratio spectra in Figure 2a, this is evident as each absorption minima is symmetric with a relatively narrow broadening. The energy of each transition is extracted and plotted in Figure 2c as a function of the magnetic field. The data points were fitted with Equation (1) (solid lines) to determine the reduced effective mass μ and bandgap energy *E*${}_{g}$. It is worth noting that the larger the number of the observed Landau level transitions, the higher the accuracy of the determined parameters is. Before discussing the obtained values of μ, it is important to emphasize the applicability of Equation (1), which holds only if the high magnetic field regime is reached, i.e., the cyclotron energy $E_{c}=\frac{\hbar eB}{\mu }$ is larger than the exciton binding energy. This is certainly not the case for the 1*s* exciton, as in most 2D layered perovskites, the binding energy is of the order of hundreds of meV [15,38], while cyclotron energy *E*${}_{c}$ reaches ∼80 meV at B=70 T (μ=0.1 m${}_{e}$). Fortunately, the higher order exciton states (i.e., 2*s*) are characterized by a much smaller binding energy *E*${}_{2s}$ (*E*${}_{2s}=\frac{E_{1s}}{4}$ in the 2D hydrogen model). For the 2*s* state, the high-field limit *E*${}_{c}>E_{2s}$ can be attained, and μ can be determined using Equation (1) [2]. In Figure 2d, the cyclotron energy is plotted for several μ values, including the reduced mass of (PEA)${}_{2}$PbI${}_{4}$ (μ=0.091 m${}_{e}$ [14]). The red, solid line indicates the *E*${}_{2s}$ for (PEA)${}_{2}$PbI${}_{4}$[44]. The high-field limit is achieved when the cyclotron energy crosses the 2*s* energy, at around B=30 T for (PEA)${}_{2}$PbI${}_{4}$. However, in other compounds, for excitons with a large μ, the high-field limit to which Equation (1) applies is reached only after 100 T (e.g., for μ=0.3).

**Figure 3:**
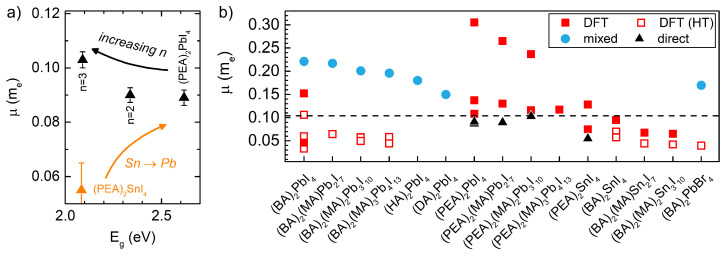
(**a**) Reduced effective masses obtained from interband magneto-absorption studies for different 2D layered perovskites. Arrows are guides to the eye. (**b**) Juxtaposition of the directly measured reduced effective mass with values obtained by various other methods (DFT-HT—DFT computed mass for the high-temperature motif). Dashed line indicates μ of (MA)PbI${}_{3}$ [10]. The DFT values from Refs. [14,49,50,51,52,53], DFT-HT values from Refs. [14,48,52,53,54,55], mixed data from Refs. [31,33,38,56], direct data from Refs. [14,15].

Figure 3a summarizes the reduced effective masses, measured to date, using interband magneto-absorption, for the 2D layered perovskites. Two trends (indicated by arrows) toward larger values of the reduced effective mass are visible and related to: (i) the Sn to Pb metal atom substitution and (ii) the increase in the number of inorganic sheets *n* in the inorganic quantum well. The substitution effect follows from the increased hybridization between the I p orbitals and the metal s orbitals in the Sn-based variants, which leads to smaller carrier masses [14]. An analogous effect was observed in the case of the 3D perovskites [13]. The influence of the number of inorganic sheets is unusual and differentiates the 2D layered perovskites from other semiconducting quantum wells. In fully inorganic quantum wells, reducing the well width increases the effective mass [45,46,47]. Such an unusual behavior for the (PEA)${}_{2}$MA${}_{n-1}$Pb${}_{n}$I${}_{3n+1}$ family is correlated with the increasing number of inorganic sheets *n*. The mass for the n=1 motif is lower than for the corresponding 3D material MAPbI${}_{3}$ (μ = 0.104 m${}_{e}$ [10]), and, with increases in the *n*, the initial 2D crystal structure approaches the bulk limit; therefore, the mass increases [15]. Interestingly, a similar trend toward higher mass values with an increasing *n* has been predicted by Stoumpos and co-workers [48] for the high-temperature motif of (BA)${}_{2}$MA${}_{n-1}$Pb${}_{n}$I${}_{3n+1}$, which, according to DFT studies, has a reduced effective mass similar to that of the PEA-based variant [14]. Nevertheless, subsequent computational studies predicted notably different mass behavior for both families [49,50]. Clearly, no consensus exists concerning how the effective mass depends on the number of inorganic sheets in 2D layered perovskites.

In Figure 3b, we compare the reduced effective masses for various 2D layered perovskites, determined from direct (magneto-optical) measurements, and for mixed experimental methods, with values obtained from the DFT band structure calculations. In the case of the DFT, the m${}_{e}$ and m${}_{h}$ were obtained from the band curvature, and, if an in-plane anisotropy was found, the mass was averaged. For the mixed experimental methods, the μ was estimated based on experimentally determined parameters, usually the diamagnetic coefficient and/or exciton binding energy. The reported μ values span a broad range from ∼0.05 to 0.3 m${}_{e}$. In all cases, the directly determined μ is lower than the value for (MA)PbI${}_{3}$ (indicated by dashed line), and the μ determined by mixed methods is larger than the value for (MA)PbI${}_{3}$. Moreover, in most cases, the DFT and DFT-HT (HT—μ computed for high-temperature motif) values lie above and below the value for (MA)PbI${}_{3}$, respectively.

## 2. Approximating the Reduced Effective Mass

The limited number of direct experimental determinations of the reduced effective mass in 2D layered perovskites found in the literature indicates how complex and experimentally challenging these measurements are. The most commonly reported parameter is the diamagnetic coefficient $c_{0}$, which is determined from magneto-spectroscopy of the 1*s* exciton state.

**Figure 4:**
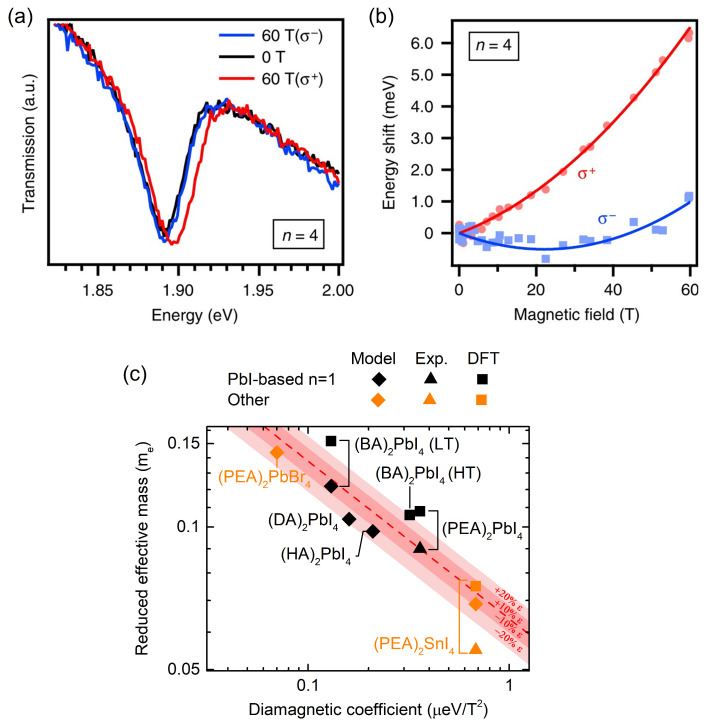
(**a**) Transmission spectra for (BA)${}_{2}$MA${}_{3}$Pb${}_{4}$I${}_{13}$ measured at B=60 T for both $\sigma^{+}$ (red) and $\sigma^{-}$ (blue) circular polarizations. (**b**) The energies of the $\sigma^{+}$ and $\sigma^{-}$ branches as a function of the magnetic field strength. Solid lines stand for fits with Equation (2). (**c**) The reduced effective mass obtained from the model described in the text (diamonds), together with the experimental [14] (triangles) and DFT-calculated [14] (squares) reduced effective masses. Dashed red line in Figure 4 is the predicted $\mu_{M}$ using Equation (3) with the parameters for (PEA)${}_{2}$PbI${}_{4}$. The shaded regions represent the boundaries corresponding to ±10 and ±20% variations in $\epsilon_{\infty }$ for (PEA)${}_{2}$PbI${}_{4}$. Panels (**a**,**b**) adapted from Blancon et al. [38].

Figure 4a shows a typical example of a polarized transmission spectrum of the 1*s* exciton of the (BA)${}_{2}$MA${}_{3}$Pb${}_{4}$I${}_{13}$ 2D perovskite [38]. The red and blue curves are $\sigma^{+}$ and $\sigma^{-}$ polarized spectra measured at B=60 T, whereas the black curve is measured without the magnetic field. The magnetic field (*B*)-induced energy shift is defined as

(2) $$\delta E_{\sigma \pm }=\pm \frac{1}{2}g\mu_{B}B+c_{0}B^{2}$$

The first term is the Zeeman splitting, where *g* is the in-plane *g*-factor and $\mu_{B}$ is the Bohr magneton, whereas the second term describes the diamagnetic shift ($c_{0}$ is the diamagnetic coefficient).

Figure 4b shows the evolution of the $E_{\sigma \pm }$ branches with an increasing magnetic field. The solid lines in Figure 4b are fits with Equation (2), from which the value of $c_{0}$ is determined. As mentioned above, to date, the value of $c_{0}$ has been determined for a number of different 2D layered perovskites. The values for the n=1 variants are summarized in Table 1.

In the hydrogen model, the diamagnetic coefficient $c_{0}\propto \epsilon_{\infty }^{2}/\mu^{3}$, where $\epsilon_{\infty }$ is the effective dielectric constant felt by the exciton (the in-plane dielectric constant $\epsilon_{\infty }^{⊥}$ or the dielectric constant normal to the magnetic field vector and the *c*-axis—see Figure 1a). By introducing an α factor into the above proportionality, i.e.,

(3) $$\mu_{M}=\alpha {\left(\frac{\epsilon_{\infty }^{2}}{c_{0}}\right)}^{\frac{1}{3}}$$

a model is formulated to approximate $\mu_{M}$ based on two input parameters: $c_{0}$ and an effective dielectric constant $\epsilon_{\infty }$ defined as

(4) $$\epsilon_{\infty }=\frac{\epsilon_{b}L_{b}+\epsilon_{w}L_{w}}{L_{b}+L_{w}}$$

where $\epsilon_{b}$ and $\epsilon_{w}$ are the dielectric constants of the organic (barrier) and inorganic (well) materials, and $L_{b}$ and $L_{w}$ are the thickness of the barrier and well layers. Using the values for (PEA)${}_{2}$PbI${}_{4}$ of $c_{0}=0.36$ μeV/T${}^{2}$ [14], μ=0.091 m${}_{e}$ [14], and $\epsilon_{\infty }$, calculated based on Equation (4), together with the data in Table 1 a proportionality factor α of 0.024 is determined.

Limiting the number of input parameters to two greatly simplifies estimation of the effective mass since the diamagnetic coefficient and the effective dielectric constant are known for many 2D layered perovskites. The latter can be also obtained from ellipsometry measurements [59] or approximated with Equation (4).

Using Equation (3), we estimated the $\mu_{M}$ values for several 2D layered perovskites, including (PEA)${}_{2}$PbBr${}_{4}$ ($\mu_{M}=$ 0.144 m${}_{e}$), (BA)${}_{2}$PbI${}_{4}$ ($\mu_{M}=$ 0.123 m${}_{e}$), and (PEA)${}_{2}$SnI${}_{4}$ ($\mu_{M}=$ 0.07 m${}_{e}$). The input values in the model are the diamagnetic coefficient and the effective dielectric constant, both taken from the published values summarized in Table 1. The $\epsilon_{w}$ for (PEA)${}_{2}$SnI${}_{4}$ and (PEA)${}_{2}$PbBr${}_{4}$ is calculated based on the $\epsilon_{\mathrm{eff}}$ ratio of their 3D counterparts and (MA)PbI${}_{3}$. For the Br-based perovskites, the $\epsilon_{\mathrm{eff}}$ ratio of (MA)PbI${}_{3}$ and (MA)PbBr${}_{3}$[11] equals $\frac{9.4}{7.5}=1.25$. Since the (PEA)${}_{2}$PbI${}_{4}$ and (PEA)${}_{2}$PbBr${}_{4}$ compounds also differ only by the halide atom, the 1.25 ratio derived for the 3D materials is used to approximate the $\epsilon_{w}$ of (PEA)${}_{2}$PbBr${}_{4}$ based on $\epsilon_{w}=6.1$ for (PEA)${}_{2}$PbI${}_{4}$. The estimated value of $\frac{6.1}{1.25}=4.88$ agrees well with the previously used $\epsilon_{w}$ of 4.8 for (BA)${}_{2}$PbBr${}_{4}$[33]. For (PEA)${}_{2}$SnI${}_{4}$, the same procedure is employed to determine an $\epsilon_{w}$ of 5.19 based on the available reports for 3D Sn-based compounds [13].

In Figure 4, the estimated values of $\mu_{M}$ are shown as a function of the diamagnetic coefficient (diamonds). If available, the experimentally determined μ, as well as the respective μ obtained from the DFT, are shown. The dashed red line in Figure 4 is the predicted variation using Equation (3) with the parameters for (PEA)${}_{2}$PbI${}_{4}$.

It is worth noting that the correct and precise assignment of the dielectric constant in 2D perovskites is a complex problem [26,38,62,63]; a multitude of reports have tackled this issue so far, and no unequivocal guidelines exist as to which value of $\epsilon_{\infty }$ should be used for 2D layered perovskites. Even for the model sample, (PEA)${}_{2}$PbI${}_{4}$, the value of $\epsilon_{\infty }$ spans a broad range. Depending on the selection of $\epsilon_{b}$, either 2.34 [64] or 3.32 [65] obtains an $\epsilon_{\infty }$ (according to Equation (4); for the full parameter list, see Table 1) equal to 3.82 or 4.41, respectively. The relative difference between these two values is ∼13%. However, the model shows that even a 20% variation in $\epsilon_{\infty }$ for (PEA)${}_{2}$PbI${}_{4}$ still provides a reasonable estimate of $\mu_{M}$. The results of the variations in $\epsilon_{\infty }$ by ±10 and ±20% are indicated in Figure 4 (shaded regions). It is worth noting that all of the estimated values are within these shaded regions, and all of the DFT data are above the respective experimental/estimated data points.

Recent studies have indicated that the diamagnetic coefficient also reflects the corrugation of the inorganic framework imposed by the organic spacers [52]. Although the organic spacers are not contributing to the conduction/valence band-edge states [66], they impose a distortion on the metal–halide network, modifying the hybridization between the respective orbitals. As a result, by simple engineering of the organic spacers, the reduced effective mass can be tuned [14]. Such a peculiar property is also captured by the model. Although L${}_{b}$ (Table 1) increases in the series L${}_{b}^{\mathrm{BA}}<$L${}_{b}^{\mathrm{HA}}<$L${}_{b}^{\mathrm{DA}}$, the effective mass does not, i.e., $\mu_{\mathrm{BA}}>$$\mu_{\mathrm{HA}}$$<\mu_{\mathrm{DA}}$, and μ follows the rather out-of-plane distortion angle $\delta_{\mathrm{BA}}>$$\delta_{\mathrm{HA}}$$<\delta_{\mathrm{DA}}$ [60,61]. The sensitivity of the diamagnetic coefficient to this peculiar property of hybrid materials indicates our model should apply to a large variety of 2D layered perovskites with different organic spacers. The dependence of μ on δ discussed above is also consistent with DFT modeling [14].

## 3. Conclusions and Outlook

The current state of research concerning the determination of the effective mass using optical methods in 2D layered perovskites was reviewed and compared with the available computational studies. It was shown that the reduced effective mass determined from a multitude of methods spans a broad range (0.05–0.3 m${}_{e}$), providing an unwelcome environment for the modeling of future opto-electronic devices based on these materials. Based on the available experimental data, we have formulated a simple scaling law for the effective mass to circumvent the above issues. The proposed model estimates the reduced effective mass using only two widely available experimental parameters, namely, the diamagnetic coefficient and the effective dielectric constant. The model has been tested on several 2D layered perovskites and correctly reproduces the main experimental trends.

Nevertheless, the estimated masses are only approximate and are subject to caution. A direct experimental verification by means of interband Landau level magneto-spectroscopy is required to verify the proposed model and provide answers to a multitude of open questions.

For example, one such question concerns the observed change in the reduced effective mass upon the phase transition in the aliphatic chain-based 2D layered perovskites [52]. Although the interband Landau levels in, i.e., (BA)${}_{2}$PbI${}_{4}$ would be difficult to observe due to the low spectral sensitivity beyond 3 eV in most high-field facilities, the Sn-based analogs ((BA)${}_{2}$SnI${}_{4}$) also exhibit a characteristic phase transition around ∼250 K and a bandgap red-shift to ∼2.1 eV [67], which greatly facilitates observation of the optical transitions between Landau levels.

Another open question concerns the μ’s dependence on the number of inorganic sheets *n* (thickness of the quantum well). Whether the scaling of μ with respect to *n* determined for (PEA)${}_{2}$(MA)${}_{n-1}$Pb${}_{n}$I${}_{3n+1}$ [15] is universal for all families of 2D layered perovskites is an ongoing debate. Additional magneto-absorption studies are urgently needed to solve this issue.

The above investigations of the n>1 structures can be extended to evaluate the dependence of μ on the corrugation of the inorganic framework. The current model suggests that the larger the distortion, the larger the reduced effective mass [14]. Here, Landau level spectroscopy of n>1 2D perovskites with different organic spacers can provide an unequivocal answer. The respective excitonic bands of these variants are red-shifted with respect to the excitons in n=1 structures, which greatly simplifies observation of the optical transitions between Landau levels.

Magneto-absorption spectroscopy involving observation of the interband Landau level transitions may also shed light on the importance of polaronic effects in 2D layered perovskites. Recently, it has been shown that these materials behave as polaronic systems [68], similar to their 3D counterparts [69]. Nevertheless, how a charge carrier’s coupling to the soft, anharmonic lattice modifies the band dispersions, and thus the effective mass, is not known. Here, the magnetic field can provide invaluable insight, as already reported for alkali halide crystals [70,71].

## Figures and Tables

**Table 1:** Parameters of selected 2D layered perovskites. From the left: diamagnetic coefficient (c${}_{0}$), high-frequency dielectric constant of organic ($\epsilon_{b}$) and inorganic ($\epsilon_{w}$) sublattices, as well as width of the organic (L${}_{b}$) and inorganic (L${}_{w}$) sublattices. $\epsilon_{w}$ for (PEA)${}_{2}$SnI${}_{4}$ and (PEA)${}_{2}$PbBr${}_{4}$ was approximated with the procedure described in the main text.

	c${}_{0}$ (μeVT${}^{-2}$)	$\epsilon_{b}$	$\epsilon_{w}$	L${}_{b}$ (nm)	L${}_{w}$ (nm)
(PEA)${}_{2}$PbI${}_{4}$	0.36 [14]	3.32 [27]	6.1 [27]	0.993 [24]	0.641 [24]
(PEA)${}_{2}$SnI${}_{4}$	0.68 [14]	3.32 [27]	5.19	0.978 [57]	0.632 [57]
(PEA)${}_{2}$PbBr${}_{4}$	0.07 [36]	3.32 [27]	4.88	1.062 [58]	0.606 [58]
(BA)${}_{2}$PbI${}_{4}$	0.13 [52]	2.1 [59]	6.5 [59]	0.693 [60]	0.618 [60]
(HA)${}_{2}$PbI${}_{4}$	0.21 [56]	2.1 [30]	6.5 [59]	0.976 [30]	0.636 [30]
(DA)${}_{2}$PbI${}_{4}$	0.16 [52]	2.44 [27]	6.5 [59]	1.499 [61]	0.641 [61]

## Data Availability

Not applicable.
